# The effectiveness of early lens extraction with intraocular lens implantation for the treatment of primary angle-closure glaucoma (EAGLE): study protocol for a randomized controlled trial

**DOI:** 10.1186/1745-6215-12-133

**Published:** 2011-05-23

**Authors:** Augusto Azuara-Blanco, Jennifer M Burr, Claire Cochran, Craig Ramsay, Luke Vale, Paul Foster, David Friedman, Zahidul Quayyum, Jimmy Lai, Winnie Nolan, Tin Aung, Paul Chew, Gladys McPherson, Alison McDonald, John Norrie

**Affiliations:** 1 The Centre for Healthcare Randomised Trials (CHaRT), Health Sciences Building, Foresterhill, University of Aberdeen, Aberdeen, AB25 2ZD, UK

## Abstract

**Background:**

Glaucoma is the leading cause of irreversible blindness. Although primary open-angle glaucoma is more common, primary angle-closure glaucoma (PACG) is more likely to result in irreversible blindness. By 2020, 5·3 million people worldwide will be blind because of PACG. The current standard care for PACG is a stepped approach of a combination of laser iridotomy surgery (to open the drainage angle) and medical treatment (to reduce intraocular pressure). If these treatments fail, glaucoma surgery (eg, trabeculectomy) is indicated. It has been proposed that, because the lens of the eye plays a major role in the mechanisms leading to PACG, early clear lens extraction will improve glaucoma control by opening the drainage angle. This procedure might reduce the need for drugs and glaucoma surgery, maintain good visual acuity, and improve quality of life compared with standard care.

EAGLE aims to evaluate whether early lens extraction improves patient-reported, clinical outcomes, and cost-effectiveness, compared with standard care.

**Methods/Design:**

EAGLE is a multicentre pragmatic randomized trial. All people presenting to the recruitment centres in the UK and east Asia with newly diagnosed PACG and who are at least 50 years old are eligible.

The primary outcomes are EQ-5D, intraocular pressure, and incremental cost per quality adjusted life year (QALY) gained. Other outcomes are: vision and glaucoma-specific patient-reported outcomes, visual acuity, visual field, angle closure, number of medications, additional surgery (e.g., trabeculectomy), costs to the health services and patients, and adverse events.

A single main analysis will be done at the end of the trial, after three years of follow-up. The analysis will be based on all participants as randomized (intention to treat). 400 participants (200 in each group) will be recruited, to have 90% power at 5% significance level to detect a difference in EQ-5D score between the two groups of 0·05, and a mean difference in intraocular pressure of 1·75 mm Hg. The study will have 80% power to detect a difference of 15% in the glaucoma surgery rate. Trial Registration: ISRCTN44464607.

## Background

The World Health Organization ranks glaucoma as the second most common cause of blindness after cataract, and as the leading cause of irreversible blindness. There are two types of glaucoma: open angle and angle closure. Although primary open angle glaucoma is the more common, primary angle closure glaucoma (PACG) is the more severe (more likely to result in irreversible blindness if not properly treated) [1]. By 2020 PACG will affect 20 million people, and 5.3 million will be blind [2]. In the UK, PACG affects between 50,000 and 100,000 people, but is estimated to cause 1000 people to suffer irreversible blindness every year, and many more live with the disability and reduced quality of life associated with glaucoma. PACG is more common in East Asia than the rest of the world. Older age and female gender are demographic risk factors. Having a small eye and thus hypermetropia (far-sightedness) is also an important risk factor. Blindness is costly to health care, society and individuals [3,4]. The effect of severe glaucoma on patients quality of life is profound (the utility associated with severe visual impairment is approximately half that of full health) [5]. The number of people diagnosed with PACG is predicted to increase substantially over the next few years as the result of an ageing population, increased optometric screening, and raised awareness of narrow angle pathologies among clinicians [6].

Within the eye the anterior chamber angle is located between the peripheral iris and the cornea. Within the anterior chamber angle the trabecular meshwork is responsible for aqueous humour (intraocular fluid) outflow. Primary angle closure (PAC) is characterised by contact between the peripheral iris and the trabecular meshwork (appositional closure) leading to an elevated IOP. This contact can ultimately result in a permanent (synechial) closure of the angle [7]. PACG occurs when high IOP damages the optic nerve and leads to visual loss and, if untreated or inadequately treated, blindness results. PACG occurs due to anatomic factors (such as a small eye, large lens, thick peripheral iris, anterior position of ciliary processes) within the eye. People with PAC and PACG can be symptomatic as an acute angle closure crisis, or more commonly asymptomatic.

The current standard care for PACG is a stepped approach of a combination of surgery (laser or incisional) and medical management. Initial surgery uses a laser to make a small hole in the iris (laser iridotomy [LI]) to open the drainage angle, and often eye drops are required as an adjunct to LI to further reduce the IOP. There are several types of drops used to lower IOP but prostaglandin and beta-blocker treatments are the most commonly used. If the drainage pathway is still closed after LI, alternative laser treatment whereby iris tissue is pulled away from the drainage angle, laser peripheral iridoplasty (LPI) is an option. If these first line treatments fail glaucoma surgery (e.g., trabeculectomy) is then indicated. Glaucoma surgery may fail to control the IOP, and in PACG complications are more likely (such as flat anterior chamber and malignant glaucoma) than for other types of glaucoma [8].

These standard approaches to PACG management have been noted to have variable success. A new approach to the management of patients with PACG (lens extraction by phacoemulsification) has, however, gained recent interest among specialists internationally. The lens of the eye plays a major role in the mechanisms leading to PACG including pupillary block and angle crowding. The hypothesis is that PACG could be treated by removing the lens (by phacoemulsification). For glaucoma patients with cataract, lens extraction is always required. However, in the absence of cataract, whether to extract the lens, and the timing of such intervention, remains open to debate. It is likely that many people with PACG (up to 50%) will go on to develop cataracts and require surgery due to ageing and to the effect of conventional glaucoma treatment [9], which may accelerate cataract progression but by this stage irreversible glaucoma damage and sight loss may have occurred. It is proposed that early lens extraction will improve glaucoma control by opening the drainage angle. This should reduce the need for medications and trabeculectomy, maintain good visual acuity, and improve quality of life compared with standard care. It will also improve the visual function in patients with hypermetropia (found in the majority of PACG patients), by correcting this refractive error.

A 2006 Cochrane systematic review by Friedman and colleagues [10] of lens extraction for PACG found no RCTs of lens extraction versus alternative treatment options for PACG. Two included non-randomized studies were deemed of poor quality. The authors concluded that there was not enough evidence to assess the superiority of lens extraction over other interventions to control IOP in this condition, and RCTs comparing lens extraction with alternative treatment options were required.

## Methods/Design

The following question will be addressed, primarily in terms of Quality of Life and vision as well as the intraocular pressure, stability of the disease and the safety of the interventions at three years after randomisation: For people with PACG what is the clinical and cost-effectiveness of early lens extraction surgery compared with standard care (usually laser iridotomy followed by a sequence of medical therapy and glaucoma surgery (e.g., trabeculectomy or another glaucoma operation)?

The hypotheses being tested are that those randomized to early lens extraction will have a higher EQ-5D quality of life questionnaire score (mean difference of 0.05); lower IOP (mean difference of 1.75 mmHg); and a 15% lower glaucoma surgery rate than those randomized to standard care at three years.

### Participants and eligibility

All people presenting, in the recruitment centres in the UK and East Asia, with newly diagnosed PACG. The inclusion criteria are:

• Diagnosis: either one of the following two types of patients: (1) primary angle closure glaucoma (PACG) or (2) primary angle closure (PAC) with IOP ≥ 30 mm Hg at diagnosis. Glaucoma is defined as: reproducible glaucomatous visual field (VF) defects (i.e. reproducible defect, in at least 2 consecutive visual fields, of two or more contiguous points with *P *< 0.01 loss or greater, or three or more contiguous points with *P *< 0.05 loss or greater in the pattern deviation plot, or abnormal Glaucoma Hemifield Test), or Glaucomatous optic neuropathy with localised absence of the neural rim or, cup disc ratio of 0.7 or more, or asymmetry of cup disc ratio of 0.2 or more in similar sized eyes/optic discs, and an IOP > 21 mm Hg on one or more occasions,

• Newly diagnosed, i.e., either (i) untreated or (ii) under medical treatment for six months or less;

• Angle closure (iridotrabecular contact), either appositional or synechial in 180 degrees or more. Primary angle closure with raised intraocular pressure (IOP ≥ 30 mm Hg on at least one occasion). Visual field loss and glaucomatous optic neuropathy, as defined above, are not present,

• Patient must be phakic in the affected eye(s);

• The participant shall be 50 years or older,

• Written informed consent obtained.

The exclusion criteria are:

• Advanced glaucoma in the potentially eligible eye as determined by either: (i) visual field loss (mean deviation worse than -15 dB) or (ii) cup-disc-ratio ≥ 0.9;

• Previously diagnosed acute angle closure attack in the potentially eligible eye;

• Increased surgical risk: e.g., corneal opacity, Fuch's endothelial dystrophy; pseudoexfoliation, previous vitreo-retinal surgery, not able to be positioned to undergo standard technique;

• Symptomatic cataract in either eye. Symptomatic cataract is defined as sufficient lens opacity such that one would normally recommend cataract surgery to relieve visual symptoms (i.e. if the participant did not have glaucoma one would recommend lens extraction).

• Any intraocular procedure or laser treatment, including previous cataract surgery, trabeculoplasty, gonioplasty, or laser iridotomy in the index eye;

• Axial length ≤ 19 mm (nanophthalmos);

• Secondary angle closure glaucoma;

• History of retinal ischaemia, macular oedema or wet age-related macular degeneration;

• Medically unfit for surgery or for completion of the trial.

### Recruitment Procedure

Eligible patients will be identified by an ophthalmologist (EAGLE principal local investigator or co-investigator) during their initial consultation and noted in a log book. When the patient returns to the clinic and if he/she is willing to join the study, informed consent will be obtained and physical baseline measurements taken. In addition the patient will complete the baseline study questionnaire (a Socio-demographic questionnaire, and the EAGLE patient reported outcome instrument including the NEI-VFQ25 [11], EQ-5D, GPI [5] and a Health Care Utilisation Questionnaire). Patients will then be randomized immediately during this appointment and informed about the allocated treatment following full written consent collection and baseline data completion.

### Randomisation and allocation

Participants will randomized to one of the two study groups in equal proportion using a web based randomisation application at the Study Data Centre at the Centre for Healthcare Randomized Trials (CHaRT, http://viis.abdn.ac.uk/HSRU/chart) in the Health Services Research Unit, University of Aberdeen. A backup telephone version will be available, hosted in Aberdeen. The randomisation algorithm will use gender, ethnicity, centre, diagnosis (PAC or PACG), and one or both eyes eligible as minimisation covariates [12].

#### Ethical approval

This study has been approved by the NRES North of Scotland Ethics Committee (Reference number: 08/S0802/153)

### Interventions

#### Intervention arm: lens extraction

**Figure 1:**
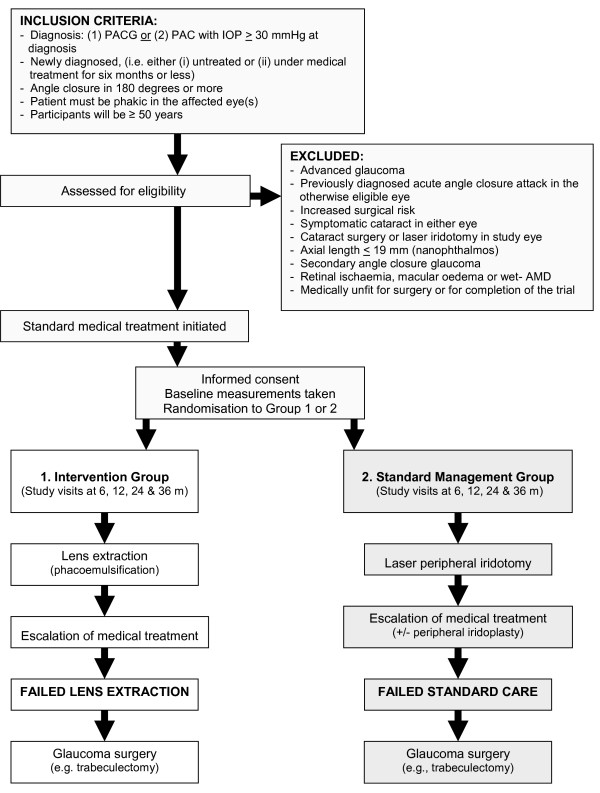
**Participants flow diagram**.

The patient will undergo phacoemulsification and intraocular lens (IOL) implant within 60 days of randomisation in the affected eye (Figure 1). A 'holding treatment' with eye drops may be started while awaiting surgery. If both eyes are eligible, the eye with more severe disease will be the first to undergo surgery, followed by the second eye within 60 days if the first intervention has been uncomplicated. If both eyes are equally affected participant choice will determine the 'index' eye. Subsequent to lens extraction surgery, if further treatment is required, the same sequence of escalation of therapy as described for 'standard care' will be used (see below). If only one eye meets the eligibility criteria this is the 'index' eye and the fellow eye will be managed according to the clinical judgement of the treating ophthalmologist and this can involve lens extraction if this is judged to be the most appropriate treatment plan.

The surgical intervention will be standardised by centres recruiting eligible surgeons to the following agreed protocol regarding preoperative measures and lens extraction:

• *Surgeon eligibility*: Fully qualified ophthalmologists who have completed the general and specialist training (in ophthalmology and glaucoma, respectively) will be able to perform lens extraction procedures.

• Le*ns extraction*: Lens extraction will be done by phacoemulsification. There is wide concordance among surgeons in the approach to lens extraction. A monofocal foldable IOL with a sharp optic edge will be inserted [13]. Synechiolysis may be done according to local practice and recorded. Intracameral cefuroxime will be used at the end of the procedure. Drops will be used post-operatively according to local practice.

1. *Topical anti-glaucoma medications*. The topical medication will consist of eye drops according to local protocol from the following list of medications-prostaglandin (OD), beta-blocker (OD), carbonic anhydrase inhibitor (BID), and alpha-2 agonist (BID), pilocarpine (QID);

2. *Escalation of topical anti-glaucoma medications*. The sequence of escalation of therapy will be: 1). single topical medication; 2). dual topical therapy; 3). triple topical therapy. Laser trabeculoplasty may be used to supplement medical therapy, according to local practice.

Glaucoma surgery will not be standardised. The type of glaucoma surgery will be chosen by the surgeon (e.g., trabeculectomy)

#### Control arm (standard care, Figure 1)

For those randomized to standard care intervention, both eyes will be treated if eligible. If only one eye meets the eligibility criteria this is the 'Index' eye and the fellow eye is managed according to the clinical judgement of the treating ophthalmologist.

Standard care will include:

1. *Laser iridotomy*: This will be the first treatment step and performed according to local protocols in each site within 60 days after randomisation. In patients with persistent angle closure (two or more quadrants) after laser iridotomy, laser peripheral iridoplasty may be done if part of local standard care;

2. *Topical anti-glaucoma medications*. The topical medication will consist of eye drops according to local protocol from the following list of medications-prostaglandin (OD), beta-blocker (OD), carbonic anhydrase inhibitor (BID), and alpha-2 agonist (BID), pilocarpine (QID);

3. *Escalation of topical anti-glaucoma medications*. The sequence of escalation of therapy will be: 1). single topical medication; 2). dual topical therapy; 3). triple topical therapy. Laser trabeculoplasty may be used to supplement medical therapy, according to local practice.

Those randomized to standard care will not cross-over to lens extraction during the study period, except for a clinical indication of cataract or only after maximum escalation of medical treatment fails to control the IOP and this would be considered as 'glaucoma surgery' (e.g., in those eyes with unresolved angle closure). Uncontrolled IOP is determined by the local ophthalmologist, and typically additional treatment would be aiming for a target IOP between 15-20 mmHg depending on the degree of optic nerve damage. After medical antiglaucoma therapy fails to control IOP, glaucoma surgery would be initiated. The need for glaucoma surgery will be qualified as a "failure" of the intervention/standard care to control the disease. Patients will remain in the trial and will continue to be followed up according to the protocol. The type of glaucoma surgery will be chosen by the surgeon (e.g., trabeculectomy).

### Safety

We aim to report serious adverse events in accordance with the guidance from the National Research Ethics Service (NRES, http://www.nres.npsa.nhs.uk) which is a subdivision of the National Patient Safety Agency http://www.npsa.nhs.uk and Good Clinical Practice (GCP).

Possible (expected) intraoperative occurrences associated with the intervention (i.e. lens extraction) are anaesthesia related, vitreous loss and need for anterior vitrectomy, iris trauma, corneal wound burn, posterior capsule rupture, lens or lens fragment loss into posterior segment; misplaced intraocular lens, zonular dialysis, supra-choroidal haemorrhage; and malignant glaucoma.

Possible (expected) occurrences associated with the intervention or with standard care, occurring at any time during the trial, include intraocular pressure spike, post-operative inflammation, posterior capsule opacification, malignant glaucoma, wound leakage, iris prolapse, IOL decentration, capsular phimosis, macular oedema, endophthalmitis, flat anterior chamber with lens-corneal touch; retinal detachment, corneal decompensation, any ocular surgical intervention for a complication, permanent loss of best-corrected visual acuity greater than 10 ETDRS letters (or two lines of Snellen chart), and systemic side effect of ocular medication requiring hospitalisation.

Details of any of the occurrences listed above will be recorded on the case report forms and reported to the Data Monitoring Committee (DMC).

### Outcome measures

The study has three primary outcomes - a patient-centred, a clinical, and an economic, reflecting the multidimensional nature of the possible effects the intervention may have. These are distinct but interrelated components of the impact of the intervention, and each component addresses a separate dimension (and so there is no issue of multiple comparisons). The study is adequately powered to address each component individually.

### Primary

*Patient-centred*: Health Status (using the EQ-5D);

*Clinical*: IOP at 3 year final assessment;

*Economic*: Incremental cost per quality adjusted life year (QALY) gained with QALYs based on the responses to the EQ-5D.

The EQ5D[14] is a multi-attribute health status classification system developed by EuroQol group. Each possible health state for each attributes of health status have an assigned value or utility. EQ5D comprises five dimensions or attribute, viz. mobility, self care, usual activity, pain/discomfort, and anxiety/depression. This instrument enables to estimate the utility from the intervention and control using the existing scoring system the responses to the questionnaire will be used to estimate health state utilities. These health state utilites estimated each time the EQ-5D is administered will be used to determine the QALY for each intervention using the area under the curve method. Annual discount rate of 3.5% will be used to discount health outcome and cost, which is based on the recommendations of the UK treasure and suggested by NICE [15].

### Secondary

#### Patient-centred

Patient reported using -

• a glaucoma specific utility instrument (GPI)^5^. This instrument is a utility-based glaucoma related health outcome measure and has been developed using a discrete choice experiment (DCE). The content of the DCE was informed by existing profile measures relevant to glaucoma to develop a preference based six-dimensional profile instrument. The dimension included were central and near vision; lighting and glare; mobility, activities of daily living; eye discomfort and other effects.

• a vision specific health profile measure (NEI-VFQ25) [11]. NEI-VFQ25 is a vision specific health profile measure based on 25-item version of National Eye Institute Visual Function Questionnaire developed in USA, and shorter version of 51 item NEI-VFQ [16]. The reliability and validity of NEI VFQ-25 were found to be comparable to those of the 51 items. NEI VFQ and suggested to be more feasible in clinical trial settings where the interview lengths needs to be shorter [11]. The multi-item subscales include items on general health, general vision, near vision, distance vision, driving, peripheral vision, colour vision, ocular pain and other vision specific like role difficulties, dependency, social functioning, and mental health.

#### Clinical

• need for glaucoma surgery (e.g., trabeculectomy); best-corrected visual acuity (ETDRS) [17]; progressive visual field loss [18]

• extension of angle closure (degrees of appositional and synechial angle closure) determined clinically; escalation of therapy; in those centres with imaging equipment the opening of the anterior chamber angle will be evaluated [19,20].

• number of anti-glaucoma medications; intolerance of medications; annual incidence of acute attacks of angle closure.

#### Economic

Costs will be based on resource use data [21,22]:

• Costs to the NHS and patients

◦ use of health services for glaucoma related events or treatments

◦ patient costs (treatments, spectacles], travel to health services, sick leave)

◦ need for alternative management for glaucoma (e.g., surgery, drugs)

◦ other use of health services

▪ visits to GP

▪ visits to practice nurse

▪ visits to optometrists

Effectiveness will be based on utility estimates from the EQ-5D and the GPI [5], and clinical outcomes [23,24]

• cost-utility analysis;

• incremental cost per QALY based on the response to the GPI [5].;

• cost-effectiveness analysis (incremental cost per case of glaucoma surgery avoided).

#### Safety

Complications during or after cataract surgery; loss of best-corrected visual acuity > 10 ETDRS letters.

The Schedule for data collection and visits is summarised in Table 1.

**Table 1:** Schedule for data collection and visits

	Baseline + Randomisation Visit month 0	Visit 1 month 6	Visit 2 month 12	Visit 3 month 24	Visit 4 month 36
Medical History	✔				

Consent/Randomisation	✔				

Humphrey Visual Fields**^1^**	✔	✔	✔	✔	✔

Pachymetry	✔				✔

Refraction	✔		✔		✔

ETDRS- Visual Acuity	✔		✔		✔

Standard clinical examination including Goldmann tonometry	✔	✔	✔	✔	✔

Gonioscopy	✔		✔		✔

Biometry	✔				

AS- OCT/UBM**^4^**	✔				✔

Participant completed baseline questionnaire (including socio - demographics, and outcome instrument)	**✔(IC)**				

Participant completed follow-up questionnaire (outcome instrument)		**✔(IC)**	**✔(IC)**	**✔(IC)**	**✔(IC)**

Health Care Utilisation Questionnaire^3^	**✔(IC)**	**✔(IC**	**✔(IC)**	**✔(IC)**	**✔(IC)**

Participant Cost Questionnaire^3^				**✔(PA)^2^**	

**Key:**

***^1 ^**If unable to perform a reliable visual field, imaging of the optic nerve is required*.

**^2 ^**Postally administrated to participants at approximately 23 months

**^3 ^**Administered in UK sites only

**^4 ^**Only for centres with this technology. AS-OCT, Anterior Segment - Optical Coherence Tomography. UBM, Ultrasound Bio-Microscopy.

***(IC) ****In clinic administration*

**(PA) **Postal administration

### Statistical analysis

A single main analysis will be performed at the end of the trial when all follow-up has been completed. Unmasked interim analyses will be conducted for the DMC meeting as determined by their agreed terms of reference. The statistical analysis will be based on all participants as randomized, irrespective of subsequent compliance with the treatment allocated.

The outcomes listed above will be compared between the experimental and control groups using generalised linear models (analysis of covariance) that adjust for the minimisation factors (and where appropriate, baseline values of outcome). Statistical significance will be at the 5% level (2P < 0.05). Analysis of co-variance will also be used in sub-group analyses performed using interaction terms (treatment group by subgroup) all at stricter levels of statistical significance (2p < 0.01). All participants will remain in their allocated group for analysis (intention to treat). Missing data statistical modelling techniques will be used to make use of outcome assessments prior to 3 years, and sensitivity analyses conducted to assess the robustness of the treatment estimates to these approaches. The type of methods that would be used include expectation maximisation algorithm, multiple imputation, general linear mixed models, techniques based on survival analysis [25]. The specific missing data approach will be pre-specified once the pattern of missingness has been determined.

The unit of analysis for the primary clinical outcome will be the eye. When a participant contributes data from two eyes the clustering will be accounted for using random effects models. For quality of life measures the unit of analysis will be the participant, with bilateral disease included as a covariate. Subgroup analysis of the primary clinical outcome (IOP) will be done according to ethnicity (Chinese or non-Chinese), and diagnosis (PAC or PACG).

We will validate the quality of life measure (NEI-VFQ25), in this patient group to assess sensitivity to change of increased visual field loss [11].

### Sample size

The primary patient reported quality of life outcome is the EQ-5D. A study with 170 participants in each group would have 90% power at 5% significance level to detect a difference in means of 0.35 of a standard deviation (SD). Our experience of using the EQ-5D in patients with moderate severity of glaucoma suggests that 0.35 SD relates to a change in EQ-5D score of 0.05 [5]. Such a mean change is likely to be both clinically and economically important. The primary clinical outcome of the study will be the comparison of IOP at 3 years post randomisation. Assuming that the standard deviation of IOP at 3 years is about 5 mmHg in both randomized groups (previous studies in PACG patients have reported standard deviations from 3.6 to 4.1 [26-28] the study would have 90% power at a 5% level of significance to detect a mean difference in IOP between the two groups of 1.75 mmHg. Previous studies based on RCT evidence in open angle glaucoma have suggested that a 2 mmHg reduction in IOP corresponds to a 20% reduction in the risk of progression [29]. We are therefore confident that the study is adequately powered to detect clinically relevant changes in IOP. The study would also have 80% power to detect a difference of 15% in the glaucoma surgery rate (assuming the glaucoma surgery rate was 40% or less). Allowing for 15% loss to follow-up at 3 years, the total number of participants required in the study is 400 (200 in each group). We have conservatively powered the study on participants, not eyes.

## Glossary of abbreviations

AMD: Age-related Macular Degeneration; AS-OCT: Anterior Segment Optical Coherence Tomography; BID: Twice a day; CHaRT: Centre for Healthcare Randomized Trials; DMC: Data Monitoring Committee; EAGLE: Effectiveness in Angle Closure Glaucoma of Lens Extraction; EQ-5D: European quality of life in 5 dimensions; ETDRS: Early Treatment Diabetic Retinopathy Study; GPI: Glaucoma Profile Index; IOL: Intraocular Lens; IOP: Intraocular Pressure; ISRCTN: International Standard Randomized Controlled Trial Number; LI: Laser Iridotomy; LPI: Laser Peripheral Iridoplasty; NEI-VFQ25: National Eye Institute Visual Function Questionnaire 25; NHS: National Health Service; NRES: National Research Ethics Service; OD: Once a day; PAC: Primary Angle Closure; PACG: Primary Angle Closure Glaucoma; QALY: Quality Adjusted Life Year; QID: Four times a day; RCT: Randomized Controlled Trial; SD: Standard Deviation; UBM: Ultrasound Biomicroscopy; UK: United Kingdom; VF: Visual Field

## Competing interests

The authors declare that they have no competing interests.

## Authors' contributions

AAB and JB: chief investigators. CC and AMcD: design of the study, standard operating procedures and data monitoring. CR and JB: design of the study, methodology. LV and ZQ: design of the study, health economic protocol. PF, DF, and WN: design of the study, clinical protocol. JL, PC, TA: design of the study, clinical protocol for Asia. GMcP: design of the protocol, website, data collection and monitoring. All authors read and approved the final manuscript.

## Funding

The study is supported by a grant from the Medical Research Council (ref G0701604).

## Previous publications

The summary protocol has been published in The Lancet [30].

## Appendix 1. Possible treatments for glaucoma (eye drops)

• CHOLINERGIC AGENTS: PILOCARPINE (TDS or QDS)

• BETA ADRENERGIC ANTAGONISTS (QD or BDS)

• ALPHA AGONISTS

• APRACLONIDINE (TDS)

• BRIMONIDINE (BDS or TDS)

• CARBONIC ANHYDRASE INHIBITORS

• DORZOLAMIDE (BDS or TDS)

• BRINZOLAMIDE (BDS or TDS)

• PROSTAGLANDINS

• LATANOPROST (QD)

• TAFLUPROST (QD)

• BIMATOPROST (QD)

• TRAVAPROST (QD)

## Appendix 2. Patient reported outcomes used in EAGLE: description, scoring and interpretation

### EQ-5D

EQ-5D is a generic health status instrument developed by EuroQOL Group that can be used for clinical and economic evaluations as well as population based surveys. EQ-5D consists of the EQ-5D descriptive system which provides a simple descriptive profile of health in five dimensions: mobility, self-care, usual activities, pain, and anxiety/depression, each with three levels. The EQ-5D also includes a visual analogue scale on which patients rate their own health between 0 (best imaginable health state) and 100 (worst imaginable health state).

A single summary index can be generated by applying a formula that attaches values to each level in each dimension. Therefore, patient's health state can be classified into one of the 243 theoretically possible health states (e.g. value of full health is 11111). These value sets are obtained using result of EQ-5D visual analogue scale valuation or the time trade-off (TTO) valuation of a representative sample of the population in several countries.

Reference for EQ-5D: The Euroqol Group. Euroqol-a faciIity for the measurement of health related quality of life. *Health policy *1990; 16:199-228. Website: http://www.euroqol.org

### NEI-VFQ 25

The NEI-VFQ 25 is a widely used vision-specific patient reported outcome measure. It measures the impact of vision problems on vision-targeted functioning and health related quality of life (HR QOL). NEI-VFQ 25 consists of 25-items with 11 subscales and a single general health rating question. A standard algorithm was used to calculate the scale scores which vary from 0 to 100 whereby 100 is the best possible score and 0 the worst. A composite NEI-VFQ 25 score can be generated by averaging the eleven scores (except the general health rating question). A higher score represents better visual functioning.

### Glaucoma Utility Index

The Glaucoma Utility Index (GUI) is a glaucoma-specific preference based (utility) measure developed for economic evaluation. GUI provides a descriptive profile in six dimensions: central and near vision, lighting and glare, mobility, activities of daily living, eye discomfort and other effects of glaucoma and its' treatment, each with four levels. A single summary index can be generated where patients' health state can be classified into one of the 4096 theoretically possible health states. Value sets are obtained by using results of discrete choice experiment (DCE) of a representative sample of glaucoma population.

## Appendix 3. Charter and responsibilities of the Data Monitoring Committee

A Data Monitoring Committee (DMC) has been established. The DMC is independent of the study organisers. During the period of recruitment to the study, interim analyses will be supplied, in strict confidence, to the DMC, together with any other analyses that the committee may request. This may include analyses of data from other comparable trials. In the light of these interim analyses, the DMC will advise the TSC if, in its view:

a) the active intervention has been proved, beyond reasonable doubt*, to be different from the control (standard management) for all or some types of participants, and

b) the evidence on the economic outcomes is sufficient to guide a decision from health care providers regarding recommendation of early lens extraction for PACG.

The TSC can then decide whether or not to modify intake to the trial. Unless this happens, however, the TSC, PMG, clinical collaborators and study office staff (except those who supply the confidential analyses) will remain ignorant of the interim results.

The frequency of interim analyses will depend on the judgement of the Chair of the DMC, in consultation with the TSC. However, we anticipate that there might be three interim analyses and one final analysis.

The Chair is Mr David Garway-Heath, with Dr David Crabb, and Professor Baljean Dhillon. Terms of reference for the DMC are available on request from the EAGLE study office.

* Appropriate criteria for proof beyond reasonable doubt cannot be specified precisely. A difference of at least three standard deviation in the interim analysis of a major endpoint may be needed to justify halting, or modifying, such a study prematurely (Peto R et al, Br J Cancer 1976;34:548-612).
